# Study of risk factors for healthcare-associated infections in acute cardiac patients using categorical principal component analysis (CATPCA)

**DOI:** 10.1038/s41598-021-03970-w

**Published:** 2022-01-07

**Authors:** Emilio Renes Carreño, Almudena Escribá Bárcena, Mercedes Catalán González, Francisco Álvarez Lerma, Mercedes Palomar Martínez, Xavier Nuvials Casals, Felisa Jaén Herreros, Juan Carlos Montejo González

**Affiliations:** 1 Intensive Care Medicine Department, Hospital Universitario 12 de Octubre, Avda. de Córdoba, s/n, 28041 Madrid, Spain; 2 Intensive Care Medicine Department, Hospital Universitario de Fuenlabrada, Fuenlabrada, Spain; 3 Intensive Care Medicine Department, Hospital del Mar, Barcelona, Spain; 4 Intensive Care Medicine Department, Hospital Universitari Arnau de Vilanova, Lleida, Spain; 5 Intensive Care Medicine Department, Vall d’Hebron Hospital Universitari, Barcelona, Spain; 6 Preventive Medicine Department, Hospital Universitario 12 de Octubre, Madrid, Spain

**Keywords:** Epidemiology, Preventive medicine

## Abstract

Using categorical principal component analysis, we aimed to determine the relationship between health care-associated infections (HAIs) and diagnostic categories (DCs) in patients with acute heart disease using data collected in the Spanish prospective ENVIN-HELICS intensive care registry over a 10-year period (2005–2015). A total of 69,876 admissions were included, of which 5597 developed HAIs. Two 2-component CATPCA models were developed. In the first model, all cases were included; the first component was determined by the duration of the invasive devices, the ICU stay, the APACHE II score and the HAIs; the second component was determined by the type of admission (medical or surgical) and by the DCs. No clear association between DCs and HAIs was found. Cronbach’s alpha was 0.899, and the variance accounted for (VAF) was 52.5%. The second model included only admissions that developed HAIs; the first component was determined by the duration of the invasive devices and the ICU stay; the second component was determined by the inflammatory response, the mortality in the ICU and the HAIs. Cronbach’s alpha value was 0.855, and VAF was 46.9%. These findings highlight the role of exposure to invasive devices in the development of HAIS in patients with acute heart disease.

## Introduction

Health care-associated infections (HAIs) are the most frequent complication in hospitalized patients^1^, with a major impact on mortality, morbidity and costs in critical care patients^2,3^. The Spanish ENVIN-HELICS registry (*Estudio Nacional de Vigilancia de la infección nosocomial en las UCI*- Hospitals in Europe Link for Infection Control through Surveillance)^4^ collects data on HAIs in Spanish ICUs and has contributed to the European HELICS registry since 2004. Of the causes of admission to ICUs contained in the ENVIN-HELICS registry, 40% of cases are for acute cardiac disease.

Traditionally, acute cardiac patients are considered to be lower risk patients for the development of HAIs compared to other critical patients, attributed, among other reasons, to the lower use of invasive devices^5–7^. However, in the last two decades, an increase has been observed in comorbidities and the incidence of noncardiac complications in such patients^8,9^. Furthermore, acute cardiac patients comprise a heterogeneous population with different subgroups or diagnostic categories (DCs) and different incidences of HAIs^10^.

The purpose of our study was to discover the relationships that exist between DCs, HAIs and other variables in patients with acute cardiac disease based on information obtained from the ENVIN-HELICS registry. For the analytical exploitation of data, a multivariant exploratory technique was employed: categorical principal component analysis (CATPCA)^11^.

## Methods

### Study sample

To conduct our study, the database of the national ENVIN-HELICS registry was used. This is a voluntary prospective registry in which the majority of Spanish ICUs participate, which collects significant information about HAIs in patients admitted for more than 24 h in the ICU between April and June. The data were obtained prospectively over a period of 10 years (from 2005 to 2015). We selected all adult patients aged over 18 whose cause of admission to the ICU was acute cardiac disease, in accordance with the categories shown in the DC variable in Table 1. The database contained a total of 175,014 admissions, of which 69,876 fulfilled this criterion.

**Table 1:** Variable definitions, category definitions and analysis level in the CATPCA models.

Variable	Included in model	Analysis level	Transformation of the original variable	Categories
Diagnostic category	1,2	Multiple nominal	None	Uncomplicated acute coronary syndrome (ACS)
				Complicated acute myocardial infarction (AMI)
				Arrhythmias (including conduction disorders)
				Heart failure (HF)
				Cardiogenic pulmonary oedema (CPE)
				Non-ACS Cardiogenic Shock (Non-ACS CS)
				Cardiac arrest (CA)
				Postoperative after cardiac surgery
				Infective endocarditis
				Miscellaneous diagnosis
HAIs	1,2	Multiple nominal	None	Ventilator-associated pneumonia (VAP)
				Catheter associated urinary tract infection@@(CA-UTI)
				Catheter related bloodstream infection (CRBSI)
				Ventilator-associated tracheobronchitis (VAT)
				Bloodstream infection secondary to other infection site (BSI-S)
				Health care-associated pneumonia (HAP)
				Urinary tract infection not associated with urinary catheter (NCA-UTI)
				Surgical site infection (SSI)
				Miscellaneous infection
				No infection (excluded in Model 2)
Medical vs. Surgical disease	1	Multiple nominal	None	Medical diagnosis
				Scheduled surgery
				Emergency surgery
Hospital Size	1	Ordinal	None	> 500 beds
				200–500 beds
				< 200 beds
Inflammatory response	2	Ordinal	None	No inflammatory response
				Sepsis
				Severe sepsis
				Septic shock
Type of admission	1	Nominal	None	Hospital admission
				Out of hospital admission
Outcome	1,2	Nominal	None	Survivor
				Non-survivor
APACHE II Score	1,2	Ordinal	Previously discretized from numerical to ordinal (octiles)	
Length of ICU stay (days)	1,2	Ordinal	Numerical to ordinal	
Duration of CVC (days)	1,2	Ordinal	Numerical to ordinal	
Duration of MV (days)	1,2	Ordinal	Numerical to ordinal	

APACHE II, “*Acute Physiology and Chronic Health disease Classification System* II”; ICU, intensive care unit; MV, mechanical ventilation; CVC, central venous catheter; ACS, acute coronary syndrome.

### Definition of the variables

Basic demographic data and the variables shown in Table 1 were included. Only HAIs acquired during the ICU stay that took place 48 h after admission were analysed.

Both the HAI definitions and the microbiological diagnostic criteria were drawn up in accordance with the criteria of the European Centre for Disease Prevention and Control (ECDC)^12^. Device-associated HAIs were included (Table 1): ventilator-associated pneumonia (VAP), catheter-associated urinary tract infection (CA-UTI), catheter-related bloodstream infection (CRBSI), and ventilator-associated tracheobronchitis (VAT)^13^. In addition, other HAIs not associated with invasive devices were included: bloodstream infection secondary to other infection sites (BSI-S), health care-associated pneumonia (HAP), urinary tract infection not associated with urinary catheters (NCA-UTI), surgical site infection (SSI), and the miscellaneous group. The inflammatory response to the infection was classified according to the Second International Sepsis Definitions Conference^14^.

### Statistical analysis

A basic descriptive analysis was conducted on the incidence of the different types of HAIs, both those associated with invasive devices and the other HAIs acquired in the ICU. Missing data were tested for randomness using Little's MCAR test.

CATPCA^11,15^ is a technique derived from linear principal component analysis (PCA) that reduces a set of numerical variables to a lower number of noncorrelated components, with the smallest loss of data possible. CATPCA performs a process known as “optimal quantification”, which allows nominal, numerical, ordinal, and categorical variables to be transformed into quantitative variables. CATPCA generates as many components as variables included in the model, although generally 2 or 3 components that contribute a greater quantity of variance are used.

In the generated graph, the transformed variables are shown as vectors in the case of numerical, ordinal, and nominal variables. The vector length or component loadings express the correlation between the component and the variable: it is an indicator of variance accounted for (VAF) and its their contribution to the component. The cosines of the angles between the vectors represent the correlation coefficient between them: an angle close to zero indicates a high correlation between variables, a 90° angle indicates no relationship and a 180° angle indicates an inverse relationship.

In the case of categorical variables, the graphic representation is produced in the form of centroids for each category. The proximity between the centroids indicates the relationship between the categories. It is possible to produce a graph that combines the vectors and the position of the centroids of the categorical variables.

To discover the relationship between DCs and HAIs, a first CATPCA model was produced for the entire sample, including a total of 10 variables (Table 1). A second model was produced including only patients who developed HAIs, which incorporated the inflammatory response to the infection and eliminated the variables related to admission. In both cases, a model with the 2 components with the greatest quantity of variance was chosen, the outliers were eliminated, and categories of low prevalence were merged. Table 1 shows the level of analysis of the variables used.

A VAF of more than 0.3 was accepted as the significant effect of a variable on the component^16^, and a Cronbach’s alpha value of more than 0.7^17^ was accepted as a measure of the internal consistency of the model.

Statistical analysis was performed with SPSS version 20.0 (IBM Corp, Armonk, NY).

### Ethics approval

The ENVIN-HELICS registry was approved by the Clinical Research Ethics Committees of the various participating hospitals: Hospital Universitario 12 de Octubre (Madrid), Hospital del Mar (Barcelona) and Hospital Arnau de Vilanova (Lleida).

### Ethics statement

The study was conducted in compliance with the current legal requirements. The ENVIN-HELICS registry was approved by the Clinical Research Ethics Committees of the various participating hospitals: Hospital Universitario 12 de Octubre (Madrid), Hospital del Mar (Barcelona) and Hospital Arnau de Vilanova (Lleida).

## Results

### Description

A total of 69,876 admissions (65.8% male) were included out of a total of 175,014 patients admitted to 231 ICUs during the study period. The average age was 66.8 years (CI 95%, 66.7 to 66.9). There were a total of 5,597 HAIs in 3616 patients; therefore, 5.1% had one or more infections (CI 95%, 5.01 to 5.34). Table 2 summarizes the incidence of the different HAIs.

**Table 2:** Incidence of health care-associated infections.

HAIs	Count (%)	Incidence per 100 admissions
**Invasive device-associated HAIs**		
VAP	1073 (28.45%)	1.54
CA-UTI	964 (25.56%)	1.38
CRBSI	699 (18.54%)	1.00
VAT	1.035 (27.45%)	1.48
All device-associated HAIs	3.771 (100%)	5.40

HAIs	Count (%)	Incidence per 100 admissions
**Other ICU-acquired HAIs**		
BSI-S	403 (22.07%)	1.55
SSI	166 (9.09%)	0.58
HAP	123 (6.74%)	0.18
NCA-UTI	53 (2.90%)	0.08
Miscellaneous infections	1081 (59.20%)	0.4
All non-device-acquired HAIs	1826 (100%)	2.6
All ICU acquired HAIs	**5597**	**8.01**
Admissions without HAIs	**66,260 (94.83%)**	
Admissions with one or more HAIs	**3616 (5.17%)**	
All admissions	**69,876 (100%)**	

HAIs, Health care-associated infections; VAP, Ventilator-associated pneumonia; CA-UTI, Catheter-related urinary tract infection; CRBSI, Catheter-related bloodstream infection; BSI-S, Bloodstream infection secondary to another infection site; VAT, Ventilator-associated tracheobronchitis; SSI, Surgical site infection; NCA-UTI, Noncatheter-related urinary tract infection HAP, Health care-associated pneumonia; ICU, Intensive Care Unit. Significant values are in [bold].

### First CATPCA model

**Figure 1:**
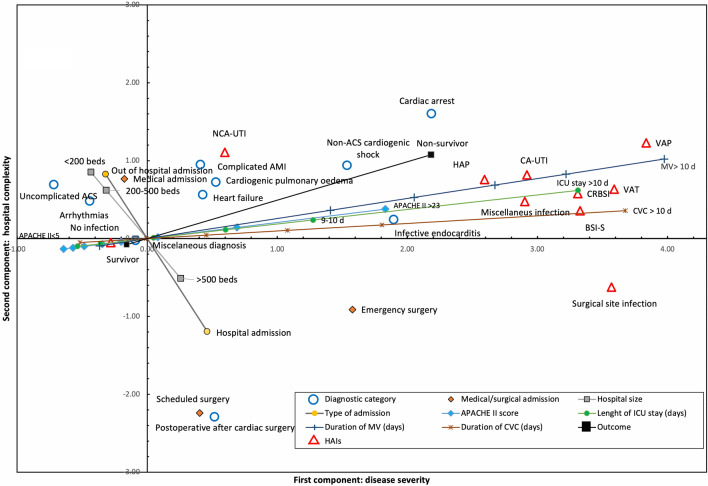
Joint representation of vectors and centroids in the first CATPCA model. Both the quantitative variables represented as vectors and the categorical variables represented as centroids were included in the joint graph of the first CATPCA model. The value of the vector end or centroid coordinates indicates the weight of the variable or category in each of the 2 components (X axis or Y axis) and the relationship to other variables. HAIs are grouped into extreme values of the first component, closely related to the duration of invasive devices and the severity of the disease (APACHE II scale and mortality). DCs have a greater weight in the second component, determined by the complexity of the hospital (medical vs. surgical patients). A DC distribution can be observed in the first component following a pattern of increasing severity. ICU: Intensive Care Unit; MV: Mechanical ventilation; CVC: Central venous catheter; APACHE II: “*Acute Physiology and Chronic Health disease Classification System* II”; HAIs: Health care-associated infections; VAP: Ventilator-associated pneumonia; CA-UTI: catheter-related urinary tract infection; NCA-UTI: Noncatheter-related urinary tract infection; CRBSI: Catheter-related bloodstream infection; BSI-S: Bloodstream infection secondary to another infection site: HAIs: Health care-associated infections; VAT: Ventilator-associated tracheobronchitis; HAP: Health care-associated pneumonia; ACS: Acute coronary syndrome; AMI Acute myocardial infarction.

The graphic result of the first model is shown in Fig. 1: it includes the vectors of the ordinal and nominal variables and the centroids of the categorical variables, making it possible to jointly evaluate the distribution and relationship of the categories of the variables in relation to the two components generated and between them.

**Figure 2:**
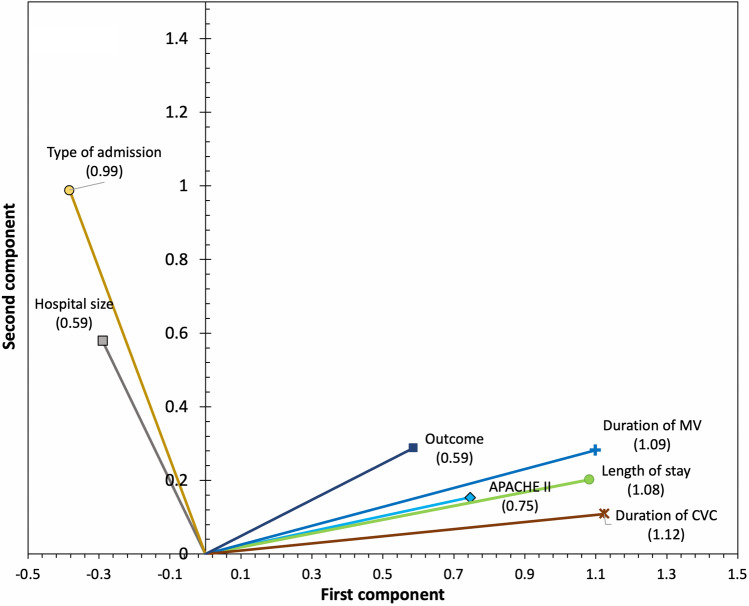
First model component loadings. The component loads indicate the correlation between the quantified variables and each of the two components, whose value is expressed by the coordinates of the end of each vector. The cosines of the angles that form the vectors indicate the correlation between variables: the variables with very close vectors are strongly related, vectors making a 90° angle indicate that variables are not related. In the first model, the variables related to the duration of invasive devices and length of stay in the ICU, the APACHE II scale and the outcome determine the first component. The type of admission and the size of the hospital determine the second component. ICU: Intensive Care Unit; MV: Mechanical ventilation; CVC: Central venous catheter; APACHE II: “*Acute Physiology and Chronic Health disease Classification System* II”.

Figure 2 allows interpretation by showing the component loadings of the ordinal variables: the variables APACHE II, length of ICU stay, duration of CVC, duration of mechanical ventilation (MV) and mortality show a high correlation with the first component, while the variables hospital size and type of admission show a high correlation with the second component.

Table 3 shows the contribution of each variable to each component: the first component is principally explained by the length of the ICU stay (VAF 0.76), duration of MV (VAF 0.78), duration of CVC (VAF 0.82) and APACHE II score (VAF 0.36). In the second component, the variables with the greatest contribution were the type of admission (VAF 0.48) and medical vs. surgical disease (VAF 0.81). Supplementary Table S1 shows the number or observations in each category.

**Table 3:** Component loadings, internal consistency and variance accounted for in the first CATPCA model.

Variable	Component loadings		Variance accounted for	
	First component	Second component	First component	Second component
Length of ICU Stay (days)	1.08	0.20	0.76	0.02
Duration of MV (days)	1.09	0.29	0.78	0.04
Duration of CVC (days)	1.12	0.19	0.82	0.006
APACHE II score	0.75	0.15	0.36	0.12
Outcome	0.59	0.29	0.22	0.04
Type of admission	− 3.83	0.99	0.09	0.48
Hospital size	− 2.94	0.59	0.06	0.17
HAIs			0.60	0.02
Diagnostic category			0.42	0.84
Medical vs. Surgical disease			0.07	0.81

Model fit statistics	First component	Second component	Total	
Cronbach’s alpha	0.85	0.66	0.90	
VAF (Multiple nominal variables)	1.10	1.68	1.38	
VAF (for non- multiple nominal variables)	3.09	0.77	3.86	
Eigenvalues	4.18	2.45	5.25	
Proportion of VAF	41.80%	24.50%	52.50%	

ICU, Intensive Care Unit; MV, Mechanical ventilation; CVC, Central venous catheter; APACHE II, “*Acute Physiology and Chronic Health disease Classification System* II”; HAIs, Health care-associated infections VAF, Variance accounted for.

DCs contribute more information to the second component (VAF 0.84) than to the first component (VAF 0.42), dividing the categories by their medical nature, situated in the upper part of the graph, or surgical nature, located in the lower part. The contribution of DCs to the first component was lower, showing a distribution with a pattern of severity: arrhythmias and uncomplicated acute coronary syndrome (ACS) were positioned in negative values, and CA, non-ACS cardiogenic shock (CS) and endocarditis were positioned in very positive values. The miscellaneous DC is located alongside the origin of both components, indicating the absence of differential characteristics.

The HAIs influence almost exclusively in the first component (VAF 0.60): the absence of infection is grouped in negative values of the first component alongside the centroids of less severe DCs, such as uncomplicated ACS and arrhythmias; the rest of the HAIs are mainly grouped in very positive values of the first component, although distant from the centroids of specific DCs.

In this model, the first component would represent the severity of the patient, and the second component would represent the complexity of the hospital. Overall, the model explains 52.50% of the total variability, with a Cronbach´s alpha value of 0.89.

### Second CATPCA model

**Figure 3:**
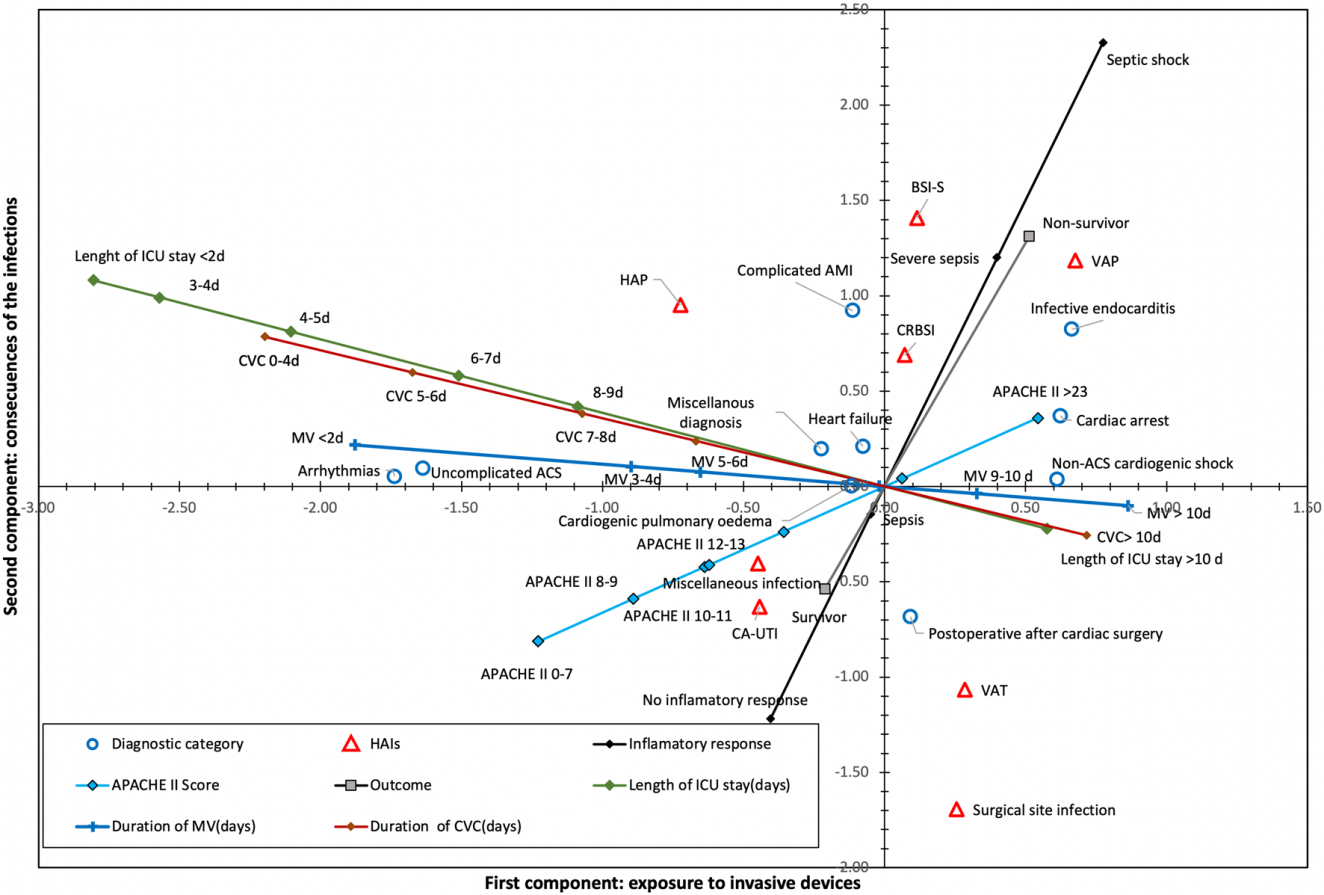
Joint representation of vectors and centroids in the second CATPCA model. The second CATPCA model includes only cases with infections. As in the first model, the duration of the invasive devices (MV, CVC), the length of the ICU stay, and the APACHE II score determine the first component (X axis). However, in this model, the inflammatory response to infection and mortality are the main determinants of the second component (Y-axis). A distribution of DC in the 2 components can be observed following a pattern of complexity and severity. HAIs are distributed in the second component with a pattern of increasing severity where the greatest weight corresponds to BSI-s and VAP. ICU: Intensive Care Unit; MV: Mechanical ventilation; CVC: Central venous catheter; APACHE II: *“Acute Physiology and Chronic Health disease Classification System* II”; HAIs: Health care-associated infections; VAP: Ventilator-associated pneumonia; CA-UTI: catheter-related urinary tract infection; NCA-UTI: Noncatheter-related urinary tract infection; CRBSI: Catheter-related bloodstream infection; BSI-S: Bloodstream infection secondary to another infection site; HAIs: Health care-associated infections; VAT: Ventilator-associated tracheobronchitis; HAP: Health care-associated pneumonia; ACS: Acute coronary syndrome; AMI: Acute myocardial infarction; CPE: Cardiogenic pulmonary oedema.

**Figure 4:**
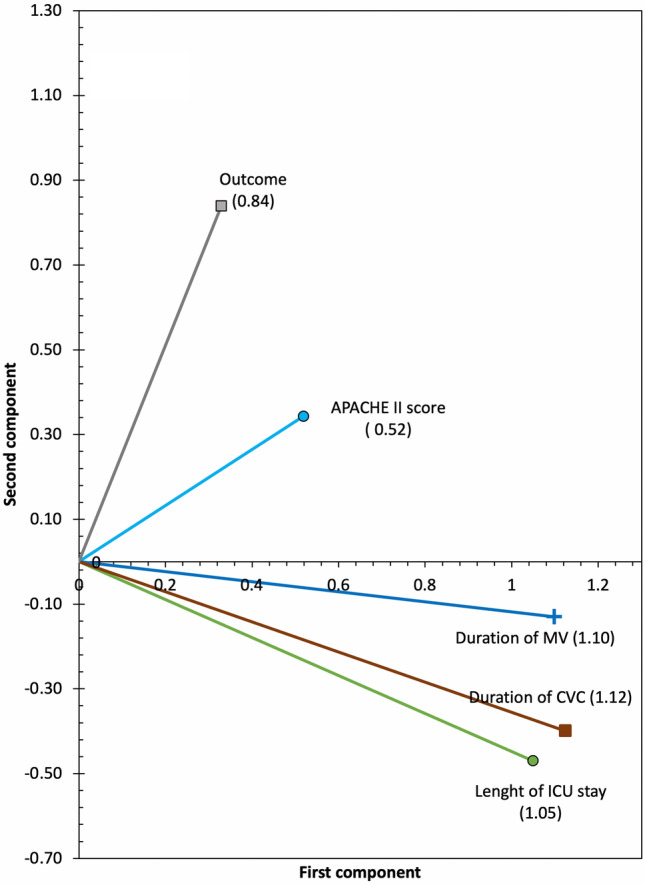
Second model component loadings. In this model, variables related to the duration of invasive devices and length of ICU stay determine the first component, while the outcome determine the second component. The APACHE II scale shows an influence on both components. ICU: Intensive Care Unit; MV: Mechanical ventilation; CVC: Central venous catheter; APACHE II: “*Acute Physiology and Chronic Health disease Classification System* II”.

Figure 3 shows the combined graph of the second model, while Fig. 4 shows the component loadings of the ordinal and nominal variables.

Table 4 shows the contribution of each variable to each component: the first component is principally explained by the variables length of ICU stay (VAF 0.67), duration of MV (VAF 0.76) and duration of CVC (VAF 0.82). In the second component, the most significant variables were inflammatory response to the infection (VAF 0.49) and mortality (VAF 0.30). Supplementary Table S2 shows the number of observations in each category.

**Table 4:** Component loadings, internal consistency and variance accounted for in the second CATPCA model.

Variable	Component loadings		Variance accounted for	
	First component	Second component	First component	Second component
Length of ICU Stay (days)	1.05	− 0.41	0.67	0.05
Duration of MV (days)	1.10	− 0.13	0.76	0.01
Duration of CVC (days)	1.12	− 0.40	0.82	0.07
APACHE II score	0.52	0.34	0.16	0.05
Outcome	0.33	0.84	0.06	0.31
Inflammatory response to infection	0.35	1.06	0.07	0.50
HAIs			0.17	0.39
Diagnostic category			0.27	0.13

Model fit statistics	First component	Second component	Total	
Cronbach’s alpha	0.75	0.39	0.85	
VAF (Multiple nominal variables)	0.40	0.50	0.48	
VAF (for non- multiple nominal variables)	2.44	1.00	3.50	
Eigenvalues	2.90	1.51	3.97	
Proportion of VAF	36.6%	18.8%	49.6%	

ICU, Intensive Care Unit; MV, Mechanical ventilation; CVC, Central venous catheter; APACHE II, “*Acute Physiology and Chronic Health disease Classification System* II”; HAIs, Health care-associated infections VAF, Variance accounted for.

The DCs contribute more information to the first component (VAF 0.27) than to the second (VAF 0.13), distributed according to the severity of the process in the horizontal axis: in negative values, arrhythmias and uncomplicated ACS, and in positive values, endocarditis, non-ACS CS and CA. It is interesting to note that DCs with a high incidence of infections, such as CA or non-ACS CS, appear in not very positive values of the second component compared to other DCs, such as endocarditis or complicated AMI, which would indicate a greater impact of HAIs on the latter.

Overall, HAIs had a greater influence in the second component (VAF 0.34) than in the first (VAF 0.17): the more acute infections were situated in positive values of the first component and above all of the second component, particularly CRBSI, VAP and BSI-S. The differentiated position of VAT compared to VAP was observed, indicating a lower mortality and inflammatory response.

The first component represents exposure to invasive devices, and the second component represents the consequences of the infection. Overall, the model explains 49% of the total variability, with a Cronbach´s alpha value of 0.85.

## Discussion

To our knowledge, this is the first study on HAIs that uses this methodology instead of the traditional statistical approach based on the inferential model. A regression analysis would be the most appropriate technique to explore the risk factors for HAIs and would provide confidence intervals for each risk factor. However, the HAI concept itself encompasses heterogeneous entities, and this would require the study of each infection individually. In our study, 3 variables of interest were qualitative variables with multiple categories, with very different sample weights, and often with artificial or irrelevant boundaries between very similar clinical entities; this makes it difficult not only to interpret the results in a regression analysis, but the preliminary descriptive analysis of these variables would be very complex as it would require multiple contingency tables with different levels. The technique used, CATPCA, makes it possible to determine nonlinear relationships between variables and to discover patterns without a pre-established hypothesis^11^. It also makes it possible to include variables of a different nature in a single model and offers us an overview of the same through its graphic representation, which makes it a very suitable tool for the study of large datasets, without the restrictions of other statistical techniques. Although rarely used in clinical studies, CATPCA is widely used in other scientific disciplines. On this point, it is worth remembering that an exploratory statistical technique was employed to study the outbreak of cholera in London in 1854^18^, 100 years before modern data mining.

The first model aims to identify the association of various risk factors and the development of HAIs in a total of 69,876 patients with cardiac diagnoses admitted to the ICU. In the model, we consider classification by DC, factors present on admission such as APACHE II (which includes factors such as age and previous comorbidities), and hospital complexity evaluated by medical-surgical character. We included the risk associated with the need for invasive devices, an essential factor in the development of HAIs^2^. The graph, which reflects the relationship established by the model between the categories of variables, distributes said categories according to their severity in the first component and medical-surgical character in the second component.

In the first component, we observe a distribution from lower to higher severity of patients, which is reflected in the APACHE II score, the DCs, the duration of the invasive devices and the development of HAIs. However, DCs and different HAIs are not associated with each other in a natural way, indicating that the development of HAIs is related more to the intrinsic severity of the patient and to the exposure to invasive devices maintained (in turn, a consequence of the severity) than to the aetiological classification on admission.

The first model has a large proportion of patients with less severe disease who do not develop HAIs. To verify whether the lack of association between HAIs and DCs is maintained, the second model limits the study to patients who presented some type of HAIs. As in the first model, the first component is principally explained by exposure to invasive devices. The second component has a different significance related to the inflammatory response to the infection and its impact on mortality.

The distribution of the infections in the first component shows a predisposition to infection associated with the devices (VAP, CRBSI) in patients with a high intrinsic severity (APACHE II greater than 20) and exposure times to devices in excess of 5–6 days. Regarding the distribution in the second component (lower vs. upper area) according to its impact in terms of inflammatory response and severity, particularly noteworthy is the low significance of ITU, VAT and SSI; in contrast, HAP, VAP, CRBSI and BSI-S are strongly associated with the development of sepsis and mortality. The distancing between VAP and VAT in the second model highlights the differences in both processes^19,20^.

The distribution of DCs is related to their intrinsic severity and therefore to exposure to invasive devices during admission. As in the first model, the surgical patient is isolated in a quadrant independent of the other categories, which indicates the convenience of treating the same as a specific entity in studies aimed at researching HAIs^21^.

There is an interesting discovery with regard to the distribution of certain DCs in relation to the prognosis: The first model places endocarditis, non-ACS CS and CA in the first component as categories with higher severity, mortality and incidence of infections. However, this perspective changes in the second model: the second component specifically expresses the prognosis associated with the development of HAIs, but CA and non-ACS CS strikingly lose the importance of the first model.

In the case of patients with CA, the high incidence of pneumonias in which bronchoaspiration plays a key role is well known^22,23^. However, premature mortality of neurological or cardiac origin would reduce the impact of HAIs.

Non-ACS CS demonstrates a similar behaviour to CA with regard to its position in the second model, which indicates that the principal determining factor of the prognosis is not infections.

Particularly noteworthy is the different behaviour of complicated AMI in the two models. The first model links complicated AMI to related categories such as heart failure (HF) and cardiogenic pulmonary oedema (CPE), distancing them from serious HAIs. However, in the second model, it is closer to HAIs with a higher inflammatory response and mortality: the HAIs show a major prognostic significance in this subgroup, which differentiates it from non-ACS CS.

The high incidence of HAIs is well documented in registries of patients with AMI^24^ and specific studies on CS after ACS^25,26^. Recent studies point to the higher severity of CS after ACS^27^ compared to non-ACS-CS, which includes patients with acutely decompensated chronic heart failure.

Both models demonstrate the importance of external factors in the development of HAIs in acute cardiac patients: exposure to invasive devices is the principal determining factor of HAIs, as shown in the VAF of those variables in the first component of both models. Increased exposure to devices in patients with a higher intrinsic severity may partially explain the development of HAIs; however, mechanisms have been suggested that favour the development of HAIs in more acute cardiac patients, such as immune paralysis^28,29^. Our study shows that DCs on admittance play a secondary role in the development of HAIs; however, it also reveals the existence of diagnostic groups with similar behaviour or, to the contrary, markedly different behaviour in terms of severity, prognosis and development of HAIs.

Our study reveals certain limitations. CATPCA is an exploratory technique in which no hypotheses are established based on a dependent variable and consequently no causal relationships are established, only associations. The ENVIN-HELICS registry is aimed at the study of HAIs in critical patients and does not contain specific information about the DCs that could be of interest, such as the existence of a category for CS owing to acute ischaemic heart disease or the performance of coronary intervention. Neither does it contain the need for circulatory support with ECMO, which is a recognized risk factor in the development of HAIs^30^.

In our study, only the APACHE II score was available. In postoperative cardiac patients, higher scores on the EUROSCORE index have been related to a greater risk of HAIs^31^; however, the role of other severity scores in nonsurgical cardiac patients^32,33^ is not well defined.

The most important clinical implication of our study is that it shows the existence of higher risk subgroups within acute cardiac patients, on which prevention measures to reduce the incidence of HAIs, such as reducing the duration of invasive devices, should be focused.

## Conclusion

By applying the CATPCA methodology in the ENVIN-HELICS registry, it is possible to obtain an overview of the factors involved in the development of HAI in acute cardiac patients, highlighting the role of exposure to invasive devices in the development of HAI and revealing the consequences of HAI in terms of severity in certain specific DCs.

## Supplementary Information

Supplementary Information.
