# Supplementary material for: Study of risk factors for healthcare-associated infections in acute cardiac patients using categorical principal component analysis (CATPCA)
Source: Sci Rep. 2022 Jan 7;12:28. doi: 10.1038/s41598-021-03970-w (PMC8742031; doi:10.1038/s41598-021-03970-w)
Supplement: Supplementary file 1 — Supplementary Information. [file 41598_2021_3970_MOESM1_ESM.docx]

**Title:**

**Health care-associated infections in acute cardiac patients: application of categorical principal component analysis (CATPCA)**

**Author’s affiliations:**

Emilio Renes Carreño, MD PhD^1^; Almudena Escribá Bárcena, MD PhD^2^; Mercedes Catalán González, MD PhD^3^; Francisco Álvarez Lerma, MD PhD^4^; Mercedes Palomar Martínez, MD PhD^5^; Xavier Nuvials Casals MD. PhD^6^; Felisa Jaén Herreros.MD.PhD^7^; Juan Carlos Montejo González, MD PhD^8^.

**1.**Intensive Care Medicine Department. “Hospital Universitario 12 de Octubre, Spain”.

**e-mail address**: [emiliorenes@gmail.com](mailto:emiliorenes@gmail.com).

ORCID: <https://orcid.org/0000-0002-3589-2438>

**2**. Intensive Care Medicine Department. “Hospital Universitario de Fuenlabrada, Spain”.

**e-mail address**: [mudesc@yahoo.es](mailto:mudesc@yahoo.es)

**3.**Intensive Care Medicine Department. “Hospital Universitario 12 de Octubre, Spain”.

**e-mail address**: mmcges@gmail.com

**4**. Intensive Care Medicine Department “Hospital del Mar, Spain”. **E-mail address:** [FAlvarez@parcdesalutmar.cat](mailto:FAlvarez@parcdesalutmar.cat)

ORCID: https://orcid.org/0000-0003-1768-3058

**5.** Intensive Care Medicine Department.” Hospital Universitari Arnau de Vilanova, Spain”. **e-mail address:** [mmpalomarmartinez@gmail.com](mailto:mmpalomarmartinez@gmail.com)

ORCID: https://orcid.org/0000-0002-6954-5645

**6**. Intensive Care Medicine Department “Vall d´Hebron Hospital Universitari, Spain**” e-mail address:** [fxnuvials@gmail.com](mailto:fxnuvials@gmail.com)

ORCID: https://orcid.org/0000-0002-6648-2394

**7.** Preventive Medicine Department “Hospital Universitario 12 de Octubre, Spain”. **e-mail address:** [felisa.jaen@salud.madrid.org](mailto:felisa.jaen@salud.madrid.org)

**8.**Intensive Care Medicine Department. “Hospital Universitario 12 de Octubre, Spain”. **e-mail address**: [jmontejohdoc@gmail.com](mailto:jmontejohdoc@gmail.com)

ORCID: https://orcid.org/0000-0002-1271-9974

**Corresponding author**: Emilio Renes Carreño, MD,PhD. Intensive Care Medicine Department. “Hospital Universitario 12 de Octubre”, Madrid, Spain. Avda. de Córdoba, s/n. Postal code 28041. Madrid, Spain. Phone: +34 913908000. Fax: +34 913908685

E-mail address: emiliorenes@gmail.com; emilio.renes@salud.madrid.org

ORCID: <https://orcid.org/0000-0002-3589-2438>

**Supplementary Table S1.** Variables included in the first CATPCA model; categories, number of observations, component loadings, and variance accounted for

|  | | | **Component Loadings** | | **VAF** | |
| --- | --- | --- | --- | --- | --- | --- |
| **Variable** | **Categories** | **N** | **First component** | **Second component** | **First component** | **Second component** |
| Medical vs. Surgical disease | Medical diagnosis | 52,143 |  |  | 0.07 | 0.81 |
|  | Scheduled surgery | 17,165 |  |  |  |  |
|  | Emergency surgery | 1,556 |  |  |  |  |
| Type of admission | Hospital admission | 29,149 | -3.83 | 0.99 | 0.09 | 0.48 |
|  | Out of hospital admission | 42,233 |  |  |  |  |
| Hospital size | > 500 beds | 40,166 | -2.94 | 0.59 | 0.06 | 0.17 |
|  | 200-500 beds | 26,910 |  |  |  |  |
|  | < 200 beds | 4,783 |  |  |  |  |
| APACHE II SCORE (Octiles) | ≤ 5 | 9,574 | 0.75 | 0.15 | 0.36 | 0.12 |
|  | 6-7 | 8,616 |  |  |  |  |
|  | 8-9 | 9,409 |  |  |  |  |
|  | 10-11 | 8,984 |  |  |  |  |
|  | 12-13 | 7,027 |  |  |  |  |
|  | 14-16 | 8,081 |  |  |  |  |
|  | 17-22 | 8,816 |  |  |  |  |
|  | ≥ 23 | 7,600 |  |  |  |  |
| Diagnostic category | Uncomplicated ACS | 28,927 |  |  | 0.42 | 0.84 |
|  | Complicated AMI | 3,830 |  |  |  |  |
|  | Arrhythmias | 8,570 |  |  |  |  |
|  | Heart failure | 2,328 |  |  |  |  |
|  | Cardiogenic pulmonary oedema | 3,185 |  |  |  |  |
|  | Non-ACS Cardiogenic Shock | 928 |  |  |  |  |
|  | Cardiac arrest | 4,246 |  |  |  |  |
|  | Postoperative after cardiac surgery | 16,944 |  |  |  |  |
|  | Infective endocarditis | 445 |  |  |  |  |
|  | Miscellaneous diagnosis | 2,456 |  |  |  |  |
| HAIs | Ventilator-associated Pneumonia | 1,073 |  |  | 0.60 | 0.02 |
|  | CA-UTI | 964 |  |  |  |  |
|  | CRBSI | 699 |  |  |  |  |
|  | Miscellaneous infection | 1,081 |  |  |  |  |
|  | BSI-S | 403 |  |  |  |  |
|  | HAP | 123 |  |  |  |  |
|  | NCA-UTI | 53 |  |  |  |  |
|  | Surgical site infection | 166 |  |  |  |  |
|  | Ventilator-associated tracheobronchitis | 1,035 |  |  |  |  |
|  | No infection | 66,262 |  |  |  |  |
| Outcome | Survivor | 67,034 | 0.59 | 0.29 | 0.22 | 0.04 |
|  | Death | 4,825 |  |  |  |  |
| Length of ICU Stay (days) | ≤ 2 d | 32,257 | 1.08 | 0.20 | 0.76 | 0.02 |
|  | 3-4 d | 21,322 |  |  |  |  |
|  | 5-6 d | 7,454 |  |  |  |  |
|  | 7-8 d | 3,152 |  |  |  |  |
|  | 9-10 d | 1,427 |  |  |  |  |
|  | >10 d | 6,247 |  |  |  |  |
| Duration of MV (days) | ≤ 2 d | 63,997 | 1.09 | 0.29 | 0.78 | 0.04 |
|  | 3-4 d | 1,683 |  |  |  |  |
|  | 5-6 d | 1,134 |  |  |  |  |
|  | 7-8 d | 796 |  |  |  |  |
|  | 9-10 d | 525 |  |  |  |  |
|  | >10 d | 3,724 |  |  |  |  |
| Duration of CVC (days) | ≤ 2 d | 49,476 | 1.12 | 0.19 | 0.82 | 0.006 |
|  | 3-4 d | 9,490 |  |  |  |  |
|  | 5-6 d | 4,411 |  |  |  |  |
|  | 7-8 d | 2,168 |  |  |  |  |
|  | 9-10 d | 1,096 |  |  |  |  |
|  | >10 d | 5,218 |  |  |  |  |

VAF: Variance accounted for; APACHE II: “*Acute Physiology and Chronic Health disease Classification System* II”; HAIs: Health care-associated infections; ICU: Intensive Care Unit; MV: Mechanical ventilation; CVC: Central venous catheter; CA-UTI: Urinary tract infection catheter-related; CRBSI: Catheter-related bloodstream infection; BSI-S: Bloodstream infection secondary to another infection site; NCA-UTI: Urinary tract infection non-catheter-related; HAIs: Health care-associated infections; HAP: Health care-associated pneumonia; ACS: Acute coronary syndrome; AMI Acute myocardial infarction; **d**: day.

**Supplementary Table S2** Variables included in the second CATPCA model; categories, number of observations, component loadings, and variance accounted for

|  | | | **Component Loading** | | **VAF** | |
| --- | --- | --- | --- | --- | --- | --- |
| **Variable** | **Categories** | **N** | **First component** | **Second component** | **First component** | **Second component** |
| APACHE II SCORE (Octiles) | ≤ 5 | 71 | 0.52 | 0.34 | 0.16 | 0.05 |
|  | 6-7 | 137 |  |  |  |  |
|  | 8-9 | 235 |  |  |  |  |
|  | 10-11 | 360 |  |  |  |  |
|  | 12-13 | 387 |  |  |  |  |
|  | 14-16 | 708 |  |  |  |  |
|  | 17-22 | 1,472 |  |  |  |  |
|  | ≥23 | 2,064 |  |  |  |  |
| Diagnostic category | Uncomplicated ACS | 390 |  |  | 0.27 | 0.13 |
|  | Complicated AMI | 581 |  |  |  |  |
|  | Arrhythmias | 247 |  |  |  |  |
|  | Cardiac failure | 245 |  |  |  |  |
|  | Cardiogenic pulmonary oedema | 405 |  |  |  |  |
|  | Non-ACS Cardiogenic Shock | 198 |  |  |  |  |
|  | Cardiac arrest | 1,373 |  |  |  |  |
|  | Postoperative after cardiac surgery | 1,874 |  |  |  |  |
|  | Infective endocarditis | 121 |  |  |  |  |
|  | Miscellaneous diagnosis | 163 |  |  |  |  |
| Length of ICU Stay (days) | ≤ 2 d | 44 | 1.05 | -0.41 | 0.67 | 0.05 |
|  | 3-4 d | 244 |  |  |  |  |
|  | 5-6 d | 368 |  |  |  |  |
|  | 7-8 d | 371 |  |  |  |  |
|  | 9-10 d | 330 |  |  |  |  |
|  | >10 d | 4,239 |  |  |  |  |
| Duration of MV (days) | ≤ 2 d | 1,213 | 1.12 | -0.13 | 0.76 | 0.01 |
|  | 3-4 d | 292 |  |  |  |  |
|  | 5-6 d | 353 |  |  |  |  |
|  | 7-8 d | 348 |  |  |  |  |
|  | 9-10 d | 293 |  |  |  |  |
|  | >10 d | 3,098 |  |  |  |  |
| Duration of CVC (days) | ≤ 2 d | 482 | 1.12 | -0.40 | 0.82 | 0.07 |
|  | 3-4 d | 232 |  |  |  |  |
|  | 5-6 d | 366 |  |  |  |  |
|  | 7-8 d | 349 |  |  |  |  |
|  | 9-10 d | 317 |  |  |  |  |
|  | >10 d | 3,851 |  |  |  |  |
| HAIs | VAP | 1,073 |  |  | 0.17 | 0.39 |
|  | CA-UTI | 964 |  |  |  |  |
|  | CRBSI | 699 |  |  |  |  |
|  | Miscellaneous infection | 1,081 |  |  |  |  |
|  | BSI-S | 403 |  |  |  |  |
|  | HAP | 123 |  |  |  |  |
|  | NCA-UTI | 53 |  |  |  |  |
|  | SSI | 166 |  |  |  |  |
|  | VAT | 1,035 |  |  |  |  |
| Outcome | Survivor | 3,971 | 0.33 | 0.84 | 0.06 | 0.31 |
|  | Death | 1,626 |  |  |  |  |
| Inflammatory response to infection | No inflammatory response | 1,461 | 0.35 | 1.06 | 0.07 | 0.50 |
|  | Sepsis | 2,834 |  |  |  |  |
|  | Severe sepsis | 737 |  |  |  |  |
|  | Septic shock | 564 |  |  |  |  |

VAF: Variance accounted for; APACHE II: “*Acute Physiology and Chronic Health disease Classification System* II”; HAIs: Health care-associated infections; ICU: Intensive Care Unit; MV: Mechanical ventilation; CVC: Central venous catheter; VAP: Ventilator-associated pneumonia; CA-UTI: Urinary tract infection catheter-related; NCA-UTI: Urinary tract infection non-catheter-related; CRBSI: Bloodstream infection; BSI-S: Bloodstream infection secondary to another infection site HAIs: Health care-associated infections; VAT: Ventilator-associated tracheobronchitis; HAP: Health care-associated pneumonia; ACS: Acute coronary syndrome; AMI Acute myocardial infarction; d: day.
